# A spray freeze dried micropellet based formulation proof-of-concept for a yellow fever vaccine candidate

**DOI:** 10.1016/j.ejpb.2019.07.008

**Published:** 2019-09

**Authors:** Didier Clénet, Véronique Hourquet, Bertrand Woinet, Hervé Ponceblanc, Manuel Vangelisti

**Affiliations:** a Bioprocess R&D Department, Sanofi Pasteur, Marcy l’Etoile, France; b MTech Department, Sanofi Pasteur, Marcy l’Etoile, France; c Analytical Sciences BIEM, Sanofi Pasteur, Neuville s/Saône, France; d R&D Global Projects Strategy and Execution, Sanofi Pasteur, Marcy l’Etoile, France

**Keywords:** Micropellets, Freeze-drying, Formulation screening, Live-attenuated vaccine stability, SEM, scanning electron microscopy, PSD, particle size distribution, XRPD, X-ray powder diffraction, NIRS, near infrared spectroscopy, DVS, dynamic vapor sorption, CCID50, cell culture infectious dose 50, DSC, differential scanning calorimetry, Tg, glass transition temperature, vYF, yellow fever virus produced in Vero cells, CMC, carboxymethyl cellulose, rHA, recombinant human albumin, Dv,10, the maximum particle diameter below which 10% of the sample volume exists, Dv,50, the maximum particle diameter below which 50% of the sample volume exists, Dv,90, the maximum particle diameter below which 90% of the sample volume exists, ASTM, American society for testing material, RH, relative humidity, AIC, Akaike information criterion, BIC, Bayesian information criterion, 95% CI, confidence interval at 95%, RMC, relative moisture content, RSS, residual sum of squares, Q10, quantity of particles in % volume having a size lower than 10 µm, w/w, weight to weight ratio, v/v, volume to volume ratio

## Abstract

The stability of live-attenuated viruses is very challenging due to thermal sensitivity; therefore, solid form is usually required (often freeze-dried products). Micropellet technology is a lyophilization technology that has the potential to provide greater flexibility in the presentation of a given vaccine particularly in multi-dose format or in combination of different vaccines. As a novel vaccine alternative process, this spray freeze-dried (SFD) micropellet technology was evaluated using as a model a yellow fever virus produced in Vero cells (vYF).

Screening of excipients was performed in order to optimize physico-chemical properties of the micropellets. Sugar/polymer-based formulations induced high glass transition temperature (Tg), adequate breaking force and attrition resistance of the SFD micropellets. These mechanical parameters and their stability are of considerable importance for the storage, the transport but also the filling process of the SFD micropellets. By adding excipients required to best preserve virus infectivity, an optimal sugar/polymer-based formulation was selected to build micropellets containing vYF. Monodisperse and dried micropellets with a diameter of about 530 µm were obtained, exhibiting similar potency to conventional freeze-dried product in terms of vYF infectious titer when both solid forms were kept under refrigerated conditions (2–8 °C). Comparable kinetics of degradation were observed for vYF formulated in micropellets or as conventional freeze-dried product during an accelerated stability study using incubations at 25 °C and 37 °C over several weeks. The results from this investigation demonstrate the ability to formulate live-attenuated viruses in micropellets. Pharmaceutical applications of this novel vaccine solid form are discussed.

## Graphical abstract

**Figure:**
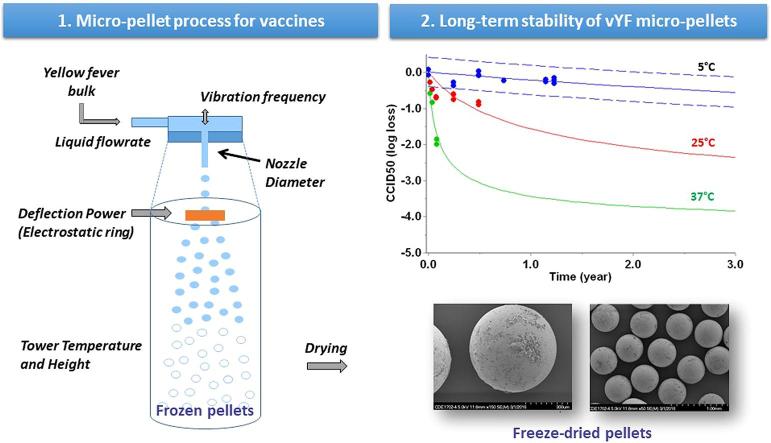

## 1 Introduction

A safe and efficacious yellow fever vaccine has existed for more than 50 years, prepared in embryonated chicken eggs and formulated as a lyophilized powder [1]. Bulk virus obtained through the embryonated eggs process is stored frozen, which then needs to be quickly processed through thawing, stabilization, and subsequent filling and lyophilization operations with minimum flexibility in order to minimize yield losses. This is a limitation when industrial scale is considered and when a need for the final bulk to be stable in liquid form before lyophilization is required.

Spray freeze-dried (SFD) micropellet technology [2] is a lyophilization technology that has the potential to provide greater flexibility in the presentation of a given vaccine particularly in multi-dose format or in combination of different vaccines. For pharmaceutical applications (monoclonal antibody), such technology was recently evaluated [3]. For vaccine, when considering multi-dose format, a customized solution can be developed from one dose to 10 doses, or even more; this can be beneficial to limit wastage in the field of vaccination campaigns. Considering combinations, several vaccines in micropellet form can be stored at the micropellet bulk stage and be combined in a single container at adapted doses thanks to an appropriate filling and packing strategy. Taking all factors into consideration, more cost-effective novel combinations can be contemplated. Proof of concept for this SFD micropellet technology with key vaccines is missing today.

A new yellow fever bulk vaccine produced in Vero cells (vYF) was developed by Sanofi Pasteur and used as a candidate in this study. The bulk virus obtained after culture is processed through purification steps, stabilized and stored in frozen form with sufficient stability in liquid form to be processed with SFD micropellet technology.

Due to the thermal sensitivity of live-attenuated virus-based vaccines, the challenges are to develop a dry solid form with low residual moisture content, which is easy to reconstitute, and that stabilizes at usual temperatures (refrigerated storage condition) against virus degradation but also physical degradation. Moreover, the final product has to possess appropriate mechanical properties (such as high breaking force and low friability) for storage and transport, and also size homogeneity to ensure good product flowability and dose uniformity compliance.

The present study was conducted to assess a proof-of-concept formulation and process of micropellets for vaccines using a yellow fever virus produced in Vero cells (vYF) as the model. First, various sugar/polymer combinations were evaluated in absence of virus for such solid form to improve their physico-chemical properties (glass transition temperature, attrition resistance, breaking force of micropellets). Second, vYF was introduced with additional excipients showing a stabilizing effect for viruses. Finally, an accelerated stability plan was performed including the same selected formulation to compare SFD micropellet with conventional lyophilized forms in terms of virus infectious titer and long-term stability predictions.

## 2 Material and methods

### 2.1 Active and excipients

#### 2.1.1 Attenuated yellow fever virus

The Yellow Fever 17D204 strain [1] was adapted to Vero cells and produced under serum-free conditions at an industrial scale (Sanofi Pasteur, Marcy L’Etoile, France). Industrial scale for manufacturing the new yellow fever vaccine can vary from 10 L to 1000 L. The virus bulk obtained after culture was processed through purification steps, stabilized at pH 8.0 in Tris buffer containing excipients (amino acids, sugar, surfactant, etc.), and stored at −70 °C in frozen form with sufficient stability in liquid form to be processed with spray freeze dried micropellet technology. The bulk was thawed overnight under refrigerated conditions before use.

#### 2.1.2 Excipients and formulations

Sucrose, sodium and carboxymethyl cellulose (CMC) were obtained from Acros (Geel, Belgium), trehalose from Pfanstiehl (Zug, Switzerland). Anhydrous potassium monophosphate, urea, maltose, Tris, L-arginine, dextran 10, Ficoll 400 and maltodextrin were obtained from Merck (Darmstadt, Germany), raffinose from Alfa Aesar (Lancashire, UK), and Povidone C-17 (PVP 10) from Ashland (Covington, USA). L-Lysine, monohydrate was obtained from Evonik (Essen, Germany). Sodium chloride dihydrate and calcium chloride were obtained from VWR (Leuven, Belgium). Sorbitol was obtained from Roquette Frères (Lestrem, France). L-Proline was obtained from Fragon (Rotterdam, The Netherlands), Poloxamer 407 sample was obtained from BASF (Ludwigshafen, Germany). Recombinant human serum albumin (rHA) was obtained from Novozymes (Franklinton, NC, U.S.A.). For formulations containing vYF, all excipients were compatible with parenteral route with a GMP grade. Based on preliminary experiments, a solid content of 25% (w/w) was selected, aiming to reach acceptable mechanical properties of SFD micropellets. All formulations tested in this study were prepared by mixing excipients with the target to reach this standardized common solid content.

### 2.2 Process

#### 2.2.1 Prilling

**Fig. 1:**
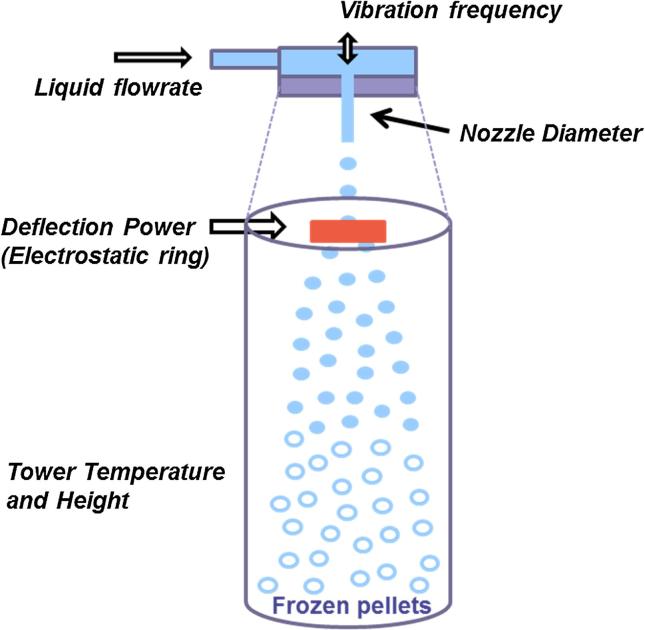
Prilling illustration indicating main process parameters for micropellets.

The 1 L scale prilling equipment comprises a prilling nozzle and a prilling tower (Fig. 1). The IE-50R prilling nozzle (INOTECH Biotechnologies Ltd., Basel, Switzerland), orifice size 300 µm, is equipped with a vibrating system to generate calibrated droplets and with an electrostatic ring to deflect them. Calibrated droplet generation is governed by the principle of controlled laminar jet break-up; the liquid is forced to cross the orifice at a constant flow rate (laminar flow), generating a liquid jet immediately out of the orifice. The vibration of the nozzle at a defined frequency induces a regular jet breaking leading to calibrated droplets. According to the Weber equation, there is a proportional relationship between the flow rate and the frequency to generate calibrated droplets (Eq. (1)) [4], [5]. These droplets cross the deflection ring to become electrically charged and, consequently, are deflected when falling in the tower. This deflection avoids any droplet coalescence and improves the cooling efficiency of the droplets. The prilling tower (Glatt, Binzen, Germany), 160 cm dropping height, is cooled by direct injection through nozzles of liquid nitrogen that is vaporized in the falling zone. The lower part of the tower has a funnel shape to recover the prills in a collector. The collector is kept at low temperature by another direct liquid nitrogen injection system to keep the prills frozen. During their fall, the droplets cool and completely freeze before touching the funnel.

(1) $$f_{\mathrm{opt}}=V^{\prime }\frac{2\sqrt{2}-1}{\pi^{2}D^{3}}\sqrt{1+\frac{3\eta }{\sqrt{\rho \sigma D}}}$$

f_opt_, optimal vibration frequency (1/s); V′, liquid flow rate (m^3^/s); D, orifice diameter of the nozzle (m); η, dynamic viscosity (Pa s); σ, surface tension (N/m); ρ, liquid density (kg/m^3^).

For a typical prilling cycle the process parameters are fixed as follows: (i) falling zone of the prilling tower at around −120 °C (temperature homogeneity ±3 °C); (ii) collector area of the prilling tower at around −90 °C; (iii) flow rate at about 16 mL/min; (iv) vibration frequency at about 2700 Hz in accordance with Weber theory and product characteristics (viscosity, surface tension and liquid density), the vibration frequency could be experimentally fine-tuned depending on the jet quality of the jet observed during the check phase before prilling phase could start in the tower; and (v) deflection power at about 1.5 kV.

#### 2.2.2 Freeze drying

The freeze dryer SMH90 from Usifroid (Elancourt, France) was used to dry the frozen micropellets, performed in batch condition on stainless-steel trays. The freeze-dryer was pre-cooled at −50 °C with the empty trays on the shelves. The frozen prills were then quickly poured as a monolayer onto the cold trays. The freeze-drying cycle takes about 20 h and mainly consists on a progressive shelf heating from −35 °C to +30 °C in 6 h, followed by a plateau of about 8 h, all steps were performed under a pressure of 50 µbar. After vacuum breaking, the free-flowing micropellets were quickly poured into glass vials (25 mg per vial) to limit water uptake and then stored with sealed closure and limited head space. The sampling in glass vials for testing was performed under a controlled relative humidity atmosphere ≤3% RH.

This freeze dryer is also used for conventional freeze-drying in vials. The filled (0.3 mL in neutral borosilicate 3 mL vials) and half-stopped vials were loaded on a shelf at 5 °C and frozen to −50 °C in 0.5 h. This low temperature was kept for 2 h. For the primary drying phase, the shelf temperature was increased to −45 °C in 25 h (while decreasing the chamber pressure to 0.045 mbar) and maintained when drying for 3 h. The secondary drying phase was performed by further increasing the shelf temperature to 25 °C in 58 h. while decreasing the chamber pressure to 0.015 mbar, followed by 24 h drying at 25 °C. At the end of the cycle, the vials were closed under nitrogen at 800 mbar, sealed with alu-caps and kept at 5 °C until analysis.

### 2.3 Analytical methods

#### 2.3.1 Freeze dried product and micropellet appearance

Pictures of micropellets and freeze-dried products were acquired by a device purchased from Microvision (Evry, France), which contains a reflected LED light (Advanced illumination, 440 State Garage Road, Rochester, USA) and a Sony digital camera XCD 90 (Sony Belgium, bijkantoor van Sony Europe Limited, Belgium). A software package (Histolab v.10.4.0 and Archimed v.10.4.0) was used to shoot pictures and convert them in jpeg format. Before analyses, micropellets were placed in a sealed 4 × 6 multiwell plate under dry atmosphere to prevent water adsorption.

#### 2.3.2 Scanning electron microscopy (SEM)

The external aspect of the micropellets was studied as follows. A dispersion of micropellets was spread on a specific analytical scotch tape (under dry air) and covered by a thin sputtered gold deposit at 25 °C for the conduction of electrical charges. The coated samples were viewed in a Hitachi S4700 microscope (Elexience, Verrières-le-Buisson, France) under standard microscope operating conditions at 5 kV.

For the internal aspect, the same operating procedure was applied to a dispersion of cut micropellets, in order to observe the inside. Cutting of micropellets was performed with a specific scalpel after spreading on the scotch tape, under dry air.

#### 2.3.3 Particle size distribution (PSD) by laser light diffraction and friability

Particle size distribution and friability of matrix micropellets (excipients without YF) were measured on Malvern-Panalytical (Malvern Ltd, Worcestershire, UK) MS2000 apparatus, and PSD of vYF containing micropellets were measured on a Malvern MS3000 apparatus.

For PSD measurements, the sample was dispersed in air (dry powder dispersion) with an optimized pressure to avoid breakage of micropellets. The obtained diffraction pattern was calculated mathematically using an optical model based on Fraunhofer theory, which provided the PSD, with the characteristic data (D_v,10_, D_v,50_, D_v,90_ and span value).

D_v,50_ is defined as the size (in µm) for which 50% of the particles (in volume) have a size lower than or equal to this value. This value is related with the average size of the micropellets in the sample. The percentile diameter ratio (D_v,90_ − D_v,10_)/D_v,50_, or span, was used to express polydispersity of the PSD and provided the width of the PSD.

Friability was assessed by laser light diffraction as follows. In contrast to PSD measurement where the pressure was optimized to avoid microbead breakage, for the friability measurement, the air pressure was increased (e.g., 3 bars). Under these conditions of elevated pressure, breakage and fines occurred, and a deformed PSD was obtained. The quantification of the fines was given by Q10 (quantity of particles in % volume having a size lower than 10 µm). The PSD was widened, which increased the Span value. The increase of Q10 and/or of the Span value was related to friability of the micropellets. These parameters were used for ranking the friability of the different micropellet formulations. Accuracy of percentile diameters was aligned with acceptance criteria defined in US Pharmacopeia 429 light diffraction measurement of particle size [6], D_v,50_ ± 10%, D_v,10_ and D_v,90_ ± 15%. Q10 values were at ±5% and Span at ±10% based on experimental reproducibility results.

#### 2.3.4 Texturometer

The resistance to breaking force of the micropellets was determined on a SMS (Stable Micro Systems Ltd, Godalming, UK) TA-XT2i texturometer equipped with Texture Expert Exceed software (version 2.64).

The desired number of beads (up to 40) was arranged in a single layer on a plate with a circular orifice of 5 mm in diameter and the exact number of beads were determined using the optical microscope and counting software previously described. This method requires narrow particle size distribution otherwise it is impossible to obtain a clear maximum force to break all the beads of the sample. The breaking force, i.e., the maximum force applied to break all the beads of the sample under the action of the texturometer probe was measured. This was graphically translated into a shoulder on the trace: Force *vs* Displacement. Five measurements were made per batch (five separate preparations), varying the number of beads with each batch. The breaking force (N) was graphically represented as a function of the number of tested beads and the value retained for breaking force (N/µpellet) was determined as the slope of the straight line obtained. Values were given at ±0.03 N/µpellet based on experimental results.

#### 2.3.5 X-ray powder diffraction (XRPD)

Samples for XRPD were ground in a mortar under dry air and placed in a flat specimen holder covered with a Kapton® film.

X-ray powder diffraction analyses were performed on a Brüker-AXS (Karlsruhe, Germany) DRX D8 Advance instrument equipped with a copper anti-cathode (Kα = 1.54056 Å) in Bragg Brentano (θ = θ) configuration. A Brüker LYNXEYE™ detector was used for this study. The diffractograms were recorded using an angular range from 3° to 80°2θ, a step of 0.02°2θ and an accumulation time per step equal to 0.5 s.

#### 2.3.6 Glass transition temperatures by differential scanning calorimeter (DSC)

Critical process temperatures namely the glass transition temperature of the maximally freeze-concentrated bulk solution surrounding the ice crystals (Tg′) and the glass transition temperature (Tg) of amorphous materials (cake, micropellets) were determined by using a power compensation DSC equipped with an Intracooler II (DSC8500; PerkinElmer LLC, Norwalk, CT, USA). Approximatively 10 µL of solution (bulk) or 2 mg of dried powder were used. The sample was sealed in an aluminum pan (with hole for powders) and an empty pan was used as reference. Cooling and heating rates of 5 °C/min were used. Liquid samples were cooled to −60 °C to ensure temperature stability and sample equilibration, and scanned for the first time to 25 °C. Tg′ determinations were done on the first heating scan. Solid samples were heated from 20 °C to 135 °C. The first scan removed residual water and the second heating scan was used to determine Tg of dried powders. Such values were used to estimate the impact of formulation compositions. All glass transition (Tg′, Tg) values were reported as the midpoint temperature of the heat capacity step associated to the glass transition. Glass transition temperatures Tg and Tg′ which were determined at ±2 °C, based on experimental reproducibility results.

#### 2.3.7 Residual moisture content by near infrared spectroscopy (NIRS)

Near infrared spectroscopy combined with chemometric method (partial least squares) was used for the determination of residual water content of micropellets and freeze‐dried formulations. A Frontier infrared spectrophotometer (Perkin Elmer LLC, Norwalk, CT, USA) was equipped with a near infrared reflectance accessory (NIRA) integrating sphere, allowing direct, non‐destructive analysis of micropellets and freeze‐dried products in vials, an near infrared source, and a separator made of calcium fluoride and a potassium bromide window. The Spectrum (version 10.5.3) and Timebase (version 3.1.4) software were used for spectra acquisition, and Spectrum Quant (version 10.4) software for construction of the model and to generate the results (quantification). A total of 87 freeze-dried samples were used to calibrate the model with thermogravimetric analysis and Karl Fischer reference values between 0.3% and 4.9 (w/w) moisture. The selected method including three principle components exhibited a variance of 99.1% and allows the determination of residual water within ±0.2% standard error. Measurements covered a spectral range from 8825 to 4000 cm^‐1^. A baseline correction with offset and standard normal variate normalization was applied to the spectra. The spectra are the result of the accumulation of eight scans, with a resolution ranging between 4 cm^−1^. The ‘interleaved’ mode was used to allow automatic background acquisition. This operation was performed on five vials (containing micropellets or a freeze-dried cake), leading to an averaged spectrum.

#### 2.3.8 Dynamic vapor sorption (DVS)

Hygroscopicity of microbeads was measured on a DVS intrinsic apparatus from Surface Measurement Systems (SMS) Ltd. (Middlesex, UK). The sample was first dried for 12 h under dry nitrogen at 25 °C and then subjected to 10% relative humidity (RH) for 24 h at 25 °C. The mass of the sample was controlled over time and the relative mass change dm/m_0_ (relative to the mass m_0_ after drying) was calculated after equilibrium was reached. Based on experimental reproducibility results, %w/w were obtained at ±0.05% when sample was maintained at 10 ± 1% RH.

#### 2.3.9 Virus titration – infectious titers

The concentration of virus was determined by a 50% cell culture infectious doses (CCID50) assay. Yellow fever virus was titrated in 96-well microtiter plates using Vero cells infected with different virus dilutions. Sample tests were diluted at a ratio of 1:4 on a serial basis (around eight dilutions) and each titration comprised ≥2 independent serial basis dilutions. Samples with high virus content were pre-diluted on a serial basis at a ratio of 1:10 to obtain the first dilution, which was to be tested on cells. After a 7–10-day incubation period at +36 °C in a 5% CO_2_ atmosphere, the number of wells presenting a cytopathic effect was determined by microscopic observation. The virus concentration was determined using a statistical method based on the least-squares method formula. The titer is expressed as CCID50/dose. Based on experimental reproducibility results, infectious titers were obtained at ±0.2 log_10_ CCID50.

#### 2.3.10 Kinetic-based modeling and stability predictions

Using forced degradation infectious titer datasets, appropriate kinetic models were developed and implemented to predict long-term stability of vYF under micropellets and freeze-dried forms. AKTS-Thermokinetics software (version 5.02, Advanced Kinetics and Technology Solutions AG (AKTS), Siders, Switzerland) was used to screen and compare kinetic models of loss of infectious titer as a function of time and temperature (5 °C, 25 °C, 37 °C), and to predict the loss of solid form infectious titer. CCID50 data obtained from freeze-dried products and micropellet samples after months of forced degradation were used to apply the modeling method. The general modeling procedure has been previously described in details [7], [8], [9]. Briefly, large variety of models from the simplest to the more complex, based on the truncated Šesták-Berggren equation were analyzed and evaluated according to a least-squares regression analysis. The kinetic parameters (A: pre-exponential factor, E: activation energy, n: reaction order, m: a parameter introduced to take into account the possible autocatalytic behavior of reaction) were systematically adjusted during the fitting procedure, comprising ‘one-step’ or ‘two-step’ models. Classical zero-order and first-order reactions, Prout-Tompkins nucleation model and derivated (Avramy-Erofeev, power law, …) and two-step models able to describe complex degradation process such as auto-accelerated reactions up to a controlled-diffusion stage (Sourour-Kamal and Finke-Watsky reactions) were tested. The best model was identified according to the higher Akaike information criterion (AIC) and Bayesian information criterion (BIC) weighted scores, and the lower sum of residual squares (RSS) value [8]. Finally, 95% confidence intervals (CIs) were calculated for predictions using bootstrap analysis (resampled 1000 times with replacement). The selected kinetic model was used to predict the course of vYF infectious titer loss during storage under isothermal conditions up to 3 years (long-term stability). The AKTS software was used to build long-term stability representations.

### 2.4 Stability monitoring by thermal stress

Micropellets and freeze-dried products were incubated in a cold chamber at 5 °C for up to 1 year and in incubators at 25 °C and 37 °C for up to 6 months. The cold chamber was maintained at ±3 °C and incubators at ±1 °C. During this forced degradation study, vYF was sampled after various times and analyzed once by biological methods (CCID50), expressed by log_10_ loss, and by physico-chemical methods (i.e. microscopy, XRPD, laser diffraction, NIRS). Solid forms were reconstituted just before analyses with 0.4% sodium chloride solution. One-year CCID50 data generated over a one-year period were used for stability modeling.

AKTS-Thermokinetics, TIBCO-Spotfire® and Excel software were used to build graphical representations.

## 3 Results

### 3.1 Screening of excipients to improve SFD micropellet properties

A single sucrose formulation presented acceptable physico-chemical properties for micropellet processability (Table 1) and was defined as a control. A monodisperse and homogeneous pellets size distribution was obtained with a diameter centered at 531 µm. Dried sucrose micropellets exhibited a breaking force of 0.3 N/pellet and a friability defined by emergence of 7% (v/v) of particles below 10 µm and Span increase of 1 when product was exposed up to 3 bars (Table 1).

**Table 1:** Main physico-chemical properties of various sugar/polymer-based micropellet formulations, without vYF. A single sucrose formulation was added as control.

		Glass transitions		Residual moisture content	Reconstitution duration	Hygroscopicity	Breaking force	Size parameters		Mechanical resistance @ 3 bars	
Formulation designations and compositions		Tg′ (°C)	Tg (°C)	RMC %(w/w)	(sec.)	%(w/w) uptake at 10%RH	(N/pellet)	Dv,50 (µm)	Span	%(v/v) part. <10 µm	Span increase
F1	10% maltose + 10% raffinose + 5% PVP 10	−26	112	1.6	29	2.3	0.3	535	0.6	12.3	1.1
F2	20% maltose + 5% maltodextrin	−27	104	1.1	18	1.8	0.4	554	0.6	9.8	0.9
F3	19.5% maltose + 0.5% CMC + 5% PVP 10	−25	101	1.1	69	1.9	0.4	544	0.6	6.9	0.8
F4	10% raffinose + 13.5% maltose + 1% maltodextrin + 0.5% CMC	−27	100	2.5	8	1.9	n. a.	584	0.6	4.8	0.3
F5	20% maltose + 5% Ficoll 400	−26	102	0.9	38	2.0	0.4	537	0.6	6.5	0.8
F6	6% maltose + 15% raffinose + 4% Ficoll 400	−26	111	1.5	25	2.1	0.4	532	0.6	4.5	0.9
F7	10% maltose + 10% raffinose + 5% Ficoll 400	−27	101	1.2	18	2.0	0.5	542	0.6	4.7	0.2
F8	20% maltose + 5% dextran 10	−25	109	1.7	10	2.0	0.5	536	0.6	3.7	0.8
F9	20% trehalose + 5% dextran 10	−26	126	2.0	6	2.5	0.6	522	0.6	3.6	0.1
F10	7.5% sucrose + 7.5% raffinose + 10% dextran 10	−22	116	0.8	10	2.4	0.5	530	0.6	3.3	0.1
F11	15% maltose + 10% dextran 10	−23	125	1.8	9	2.4	0.7	528	0.6	3.7	0.1

*Not performed.

**Fig. 2:**
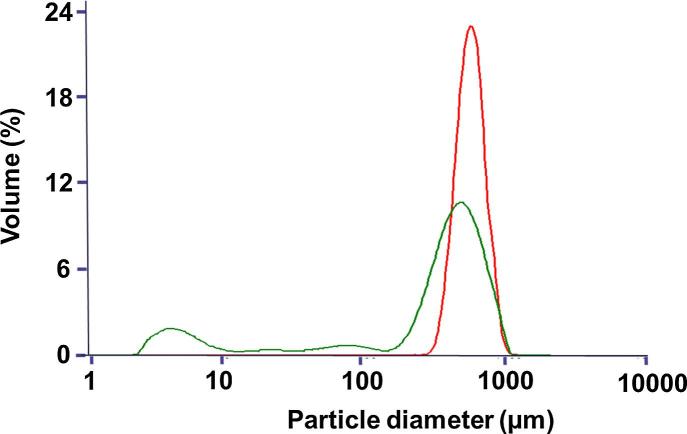
Particle size distributions of micropellets as received (red) and after a dispersion pressure from 0.8 to 3 bars in the case of a brittle formulation (green).

**Fig. 3:**
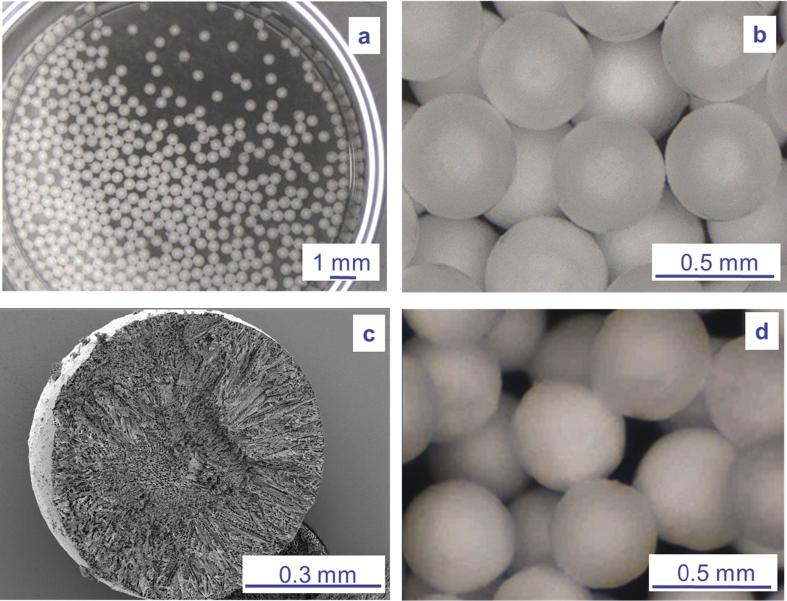
Micropellet images. (a, b) Representative micropellet appearance made with digital camera. (c) View of a representative cut micropellet by scanning electron microscopy (d) Representative micropellets made with digital camera after humidity stress, 3 days under ambient humidity.

**Fig. 4:**
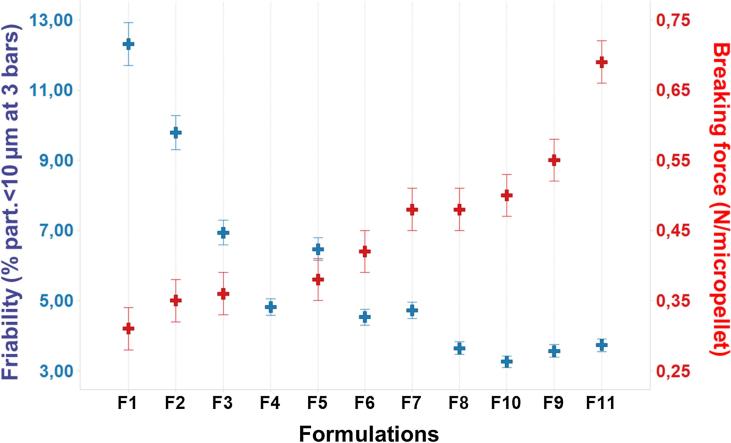
Mechanical resistance of SFD micropellets at 3 bars expressed as percentage of particles that were <10 µm and breaking force of micropellets depending on formulations (see Table 1 for detail compositions).

Mixing polymers and sugars led to formulations with Tg′ from −27 °C to −22 °C and Tg from 100 °C to 126 °C (Table 1). Aiming to improve productivity, avoid collapse phenomenon and enhance product stability, such elevated glass transitions were above acceptable internal criteria, previously defined as Tg′ > −35 °C and Tg > 50 °C. Residual moisture content values of micropellets were all below 3% (w/w) (Table 1). For all the tested mixtures, PSDs looked homogeneous with a median diameter (D_v,50_) and a polydispersity (span) at mainly 530 µm and 0.6, respectively (Table 1). In terms of morphological aspect, homogeneous pellets were observed (Fig. 3a and b) with a regular internal texture, providing porous solid beads (Fig. 3c). For micropellets that are exposed to an ambient humidity, water intake could progressively induce sticking of the pellets (Fig. 3d). Sensitivity to humidity (hygroscopicity) varied from 1.8% to 2.5% when micropellets were exposed to 10% RH. Dextran-based formulations were the most hygroscopic (Table 1). Different levels of breaking force (0.3–0.7 N/pellet) were observed depending on the formulation. Only formulation F4 induced high micropellet size polydispersity, preventing determination of breaking force (Table 1). Mechanical stress generated by an increase of pressure from 0.8 to 3 bars induced emergence of small particles below 10 µm in various proportions ranging from 3.3% (v/v) to 12.3% (v/v), depending on mixture compositions (Table 1). For the most fragile formulation (F1), pressure increase can have a strong impact on PSD with a decrease of the main population initially centered on 530 µm and the resulting PSD covering a large size range from a few µm to 1 mm after increasing the pressure (Fig. 2). In contrast, the hardest formulations containing dextran 10 and Ficoll 400 showed the best attrition resistance, i.e. lower friability, depicted by the relatively stable span value at 3 bars and low proportion of particles <10 µm (Table 1). Our results demonstrated inverse relationship between friability and breaking force of formulations (Fig. 4). Finally, reconstitution of micropellets was rapid (<1.5 min, Table 1) and all formulations were amorphous, as determined by XRPD, indicating absence of compound crystallization (data not shown) during micropellet process.

### 3.2 Proof of concept of SFD micropellets containing vYF

Various sugar/polymer-based formulations (F12 to F16) were tested to stabilize vYF virus in terms of infectious titer in micropellet form. Trehalose-based formulations were selected adding dextran 10 or PVP 10. Additional excipients including amino acids, salts, proteins, polyols and surfactants were screened; several of which negatively affect glass transitions, although mostly tested formulations exhibited Tg′ > −35 °C and Tg > 50 °C (Table 2) as expected. Note that glass transitions were highly affected by formulation compositions, leading to variation of >10 °C. In contrast, presence of additional excipients required to stabilize vYF did not significantly affected size parameters (D_v,50_, Span - Table 2) compared with previous formulations without viruses (Table 1). Residual moisture content (RMC) ranged from 1.1% to 3.0%. Infectious titer loss due to process (gap from target) ranged from 0.4 to 0.8 log_10_ CCID50 depending on the formulation (Table 2). In all the tested formulations, <1.0 log_10_ CCID50 loss was observed when the process was applied and when such solid forms were exposed at 37 °C for 1 week. F15 and F16 formulation exhibited the best protective effect. The latter was selected for further stability studies in micropellet *vs*. freeze-dried form.

**Table 2:** Main physico-chemical properties of micropellet formulations containing vYF and virus infectious titers losses (log_10_ CCID50) due to process (gap from target) and due to a thermal stress of 1 week at 37 °C (loss from t-zero).

		Glass transitions		Residual moisture content	Size parameters		Infectious titer loss (log_10_ CCID50)	
	Formulations	Tg′ (°C)	Tg (°C)	RMC %(w/w)	Dv,50 (µm)	SPAN	Gap from target	Loss after 1 week at 37 °C
F12	5.5% Trehalose + 7.5% Sucrose + essential and non-essential amino acids + 1% L-Arg + 7% Dextran 10 + 3.75% Sorbitol + 0.25% Urea	−33	37	2.2	535	0.2	−0.8	−0.8
F13	15% Trehalose + 1% rHA + 8.8% Dextran 10 + 0.2% P407	−25	75	3.0	526	0.2	−0.8	−0.8
F14	10% Trehalose + 5% Sucrose + 0.32% L-Pro + 0.03% L-Lys + 0.1% CaCl_2_ + 5.3% Dextran 10 + 4% Sorbitol + 0.25% Urea	−32	64	2.5	528	0.3	−0.8	−0.8
F15	13.6% Trehalose + 0.8% rHA + 0.1% CaCl_2_ + 2% Dextran 10 + 3% Sorbitol + 0.25% Urea	−34	72	1.2	482	0.6	−0.4	−0.9
F16	13.6% Trehalose + 0.32% L-Pro + 0.03% L-Lys + 0.1% CaCl_2_ + 2% PVP 10 + 0.2% P407 + 3% Sorbitol + 0.25% Urea	−32	56	1.1	497	0.5	−0.6	−0.6

### 3.3 Stability of vYF in SFD micropellets *vs*. freeze-dried form

**Fig. 5:**
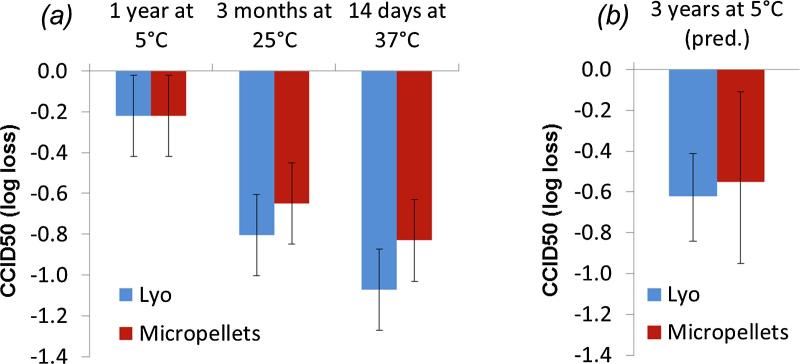
Thermal stability of F16 formulation under SFD micropellets (red) and conventional freeze-dried (blue) forms expressed as infectious titer losses (log_10_ CCID50) experimentally determined after incubations for 1 year at 5 °C, 3 months at 25 °C and 14 days at 37 °C (a). Error bars represent 95% CI based on method validation. Predictions of infectious titer losses were added for 3 years at 5 °C based on kinetic models (Eqs. (2), (3)) and error bars depicting boostrap 95% CI (b). Based on WHO/UNICEF considerations in global immunization program, ≤1.0 log_10_ loss is considered as acceptable after 14 days at 37 °C, 3 months at 25 °C and 3 years at 5 °C for live-attenuated vaccine with a medium stability [40].

For 1 year at 5 °C, vYF infectious titer remained fairly stable in F16 formulation (−0.22 ± 0.20 log_10_ CCID50 loss), similarly in either micropellet or freeze-dried form (Fig. 5a). Incubation of drug products at 25 °C and 37 °C induced loss of infectious titer. <1.0 log_10_ CCID50 loss was observed after 3 months at 25 °C and close to 1.0 log_10_ CCID50 after 14 days at 37 °C for both micropellets and freeze-dried forms (Fig. 5a).

**Fig. 6:**
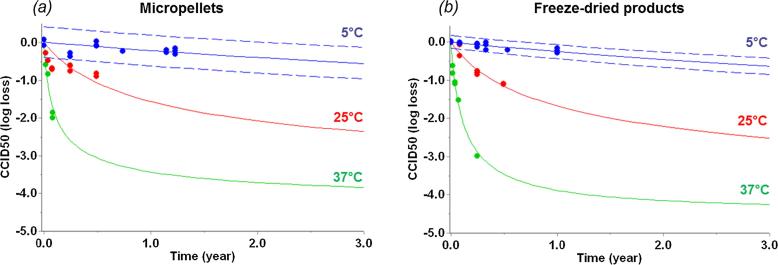
Long-term vYF infectious titer of micropellets (a) and lyophilizate (b) predicted by kinetic model (lines) at 5 °C, 25 °C and 37 °C. Data used for kinetic modeling are displayed as filled circles. At 5 °C, infectious titer prediction is shown with predictive band representing 95% CI (dotted lines).

Using such forced degradation datasets, appropriate kinetic models were developed and implemented to predict long-term stability of vYF under micropellets and freeze-dried forms. The best models were identified according to the higher Akaike information criterion (AIC) and Bayesian information criterion (BIC) weighted scores, and the lower sum of residual squares (RSS) values (Table S1). Note that simple zero-order or first-order reactions with only two adjusted kinetic parameters were not able to fit experimental data. Two-step kinetic models, including four adjusted kinetic parameters (two activation energies and two pre-exponential factors) were required to appropriately fit loss of infectious titer as a function of time and temperature (Table S1), indicating a complex reaction. The models well describing loss of infectious titer for micropellets and conventional lyophilized forms were identified as follow (Eqs. (2), (3), respectively):

(2) $$\frac{\text{d}\alpha }{\text{dt}}\text{=2.3}E\text{31.exp}\left(-\frac{\text{225.1}E\text{3}}{\text{RT}}\right).{\left(1-\alpha \right)}^{\text{3}}\text{+ 4.3}E\text{6.exp}\left(-\frac{\text{82.1}E\text{3}}{\text{RT}}\right).{\left(1-\alpha \right)}^{4}$$

(3) $$\frac{\text{d}\alpha }{\text{dt}}\text{=3.8}E\text{34.exp}\left(-\frac{\text{245.1}E\text{3}}{\text{RT}}\right).{\left(1-\alpha \right)}^{2}+3.0E\text{7.exp}\left(-\frac{\text{86.1}E\text{3}}{\text{RT}}\right).{\left(1-\alpha \right)}^{4}$$

where for example, in Eq. (3), dα/dt represents the reaction rate, 3.8E34 and 3.0E7 depict the values of the pre-exponential factors A1 and A2 for first- and second steps, 245.1E3 and 86.1E3 represent the values of the activation energy E1 and E2, exponents 2 and 4 show the value of the reaction order in the first and second step, respectively. Selected models (Eqs. (2), (3)) were used to simulate the vYF infectious titer stability over 3 years at 5 °C (Fig. 6). Predictive bands at 5 °C were larger for micropellets with bootstrap 95% confidence intervals at ±0.5 log_10_, compared to ±0.2 log_10_ for lyophilized products (Fig. 6, dashed lines). Similar stable infectious titer was predicted for both micropellets and lyophilizates when stored at 5 °C, reaching −0.55 and −0.62 log_10_ losses after 3 years, respectively (Fig. 5b). Following this kinetic-based modeling approach, <1.0 log_10_ CCID50 loss is expected after 3 years at 5 °C for both solid forms. Taking into account predictive bands (bootstrap 95% CI), vYF infectious titer should be from −0.95 and −0.11 log_10_ loss and from −0.84 and −0.41 log_10_ loss after 3 years at 5 °C for both micropellets and lyophilizates, respectively (Figs. 5b and 6). Both solid forms would be unstable under higher storage temperatures. A gradual decrease in infectious titer over time was predicted at 25 °C, with a mean decrease to −1.8 log_10_ at 2 years (Fig. 6). Rapid loss in infectious titer was predicted at 37 °C incubation temperature, with >3 log_10_ loss after 2 years.

In terms of micropellet physical properties, size and aspect remained unchanged after 1 month at 25 °C and 37 °C and 1 year at 5 °C with an average diameter at around 500 µm (data not shown). Furthermore, micropellets were still amorphous, indicating absence of excipient crystallization during recommended storage conditions.

## 4 Discussion

To reduce cooperative molecular mobility in amorphous solids and improve long-term stability of bioproducts, a convenient way is to increase Tg by introducing polymers. For this work, a list of pharmaceutical additives to examine was chosen using scientific rationale based on literature review [10], [11], [12], [13], [14] and pharmaceutical experience. Sugars selected as bulking agent for this study were sucrose, maltose, trehalose and raffinose. As determined by DSC, they exhibit Tg′ values between −35 °C and −27 °C [12], [15], [16] and Tg values between 43 °C and 115 °C [17], [18], [19]. To enhance glass transitions, polymer can advantageously be added in sugar-based formulations [20]. Polymers showed elevated Tg values, often above 90 °C and can advantageously be used to enhance glassy state stability of drug products [17], [19], [20], [21]. With high Tg′, higher than −22 °C, dextran 10, PVP 10, Ficoll 400 and maltodextrin were identified as candidates of interest [10], [12], [16]. Sodium carboxymethyl cellulose was also evaluated because of its well-known high Tg value. In agreement with a previous study [20], results presented in this paper demonstrate that addition of a polymer in sugar-based formulations drastically increases glass transition temperatures.

Finally, for initial process feasibility, a viscosity below 5 mPa s and a solid content of 25% (w/w) were chosen, knowing that such value looked acceptable in terms of micropellet processability, as previously evaluated with 20% and 30% sucrose micropellets.

A homogeneous PSD was obtained, around 530 µm independently of tested formulations. The size of micropellets directly depends on the orifice diameter of the nozzle and is governed by the laminar liquid-jet break-up principle. The rationale for targeting the size of the micropellets is driven by a compromise with i) a size big enough for an easy product handling (weak electrostatic behavior) (ii) a size small enough to limit equipment sizing (height of the prilling tower).

The combination of a polymer (mainly dextran 10 or Ficoll 400) with a sugar (sucrose, maltose, trehalose or raffinose) also showed a positive effect on mechanical properties. Indeed, except for two formulations (F1 and F2), mixtures of sugars and polymers reduced friability of micropellets as shown by low proportion of particles <10 µm and by moderate change in Span when products were exposed up to 3 bars (Table 1). Correlatively these sugar/polymer-based formulations can also induce a significant increase of micropellet breaking force (Table 1). The effect is significantly higher for dextran-based formulation (F8 to F11) that can be identified as candidates of interest. In the pharmaceutical industry, water-soluble polymers such as dextran are used to provide mechanical strength to tablets (binder) [22]. Dextran is also used during lyophilization as a bulking agent and/or a collapse temperature modifier. This excipient was recently included in the lyophilized antibody–drug conjugate product MYLOTARG® (gentuzumab ozogamicin) [11]. In spite of the dextran 10 sensitivity to humidity (see hygroscopicity for F8 to F11 in Table 1), residual moisture contents of micropellets containing dextran 10 were below 3% (w/w), an acceptable level based on regulatory guidelines [23]. However, knowing that micropellets offer larger geometrical exposed area compared with freeze-dried form, enhanced sensibility to environmental humidity is a critical parameter to take into account by optimizing process parameters and handling conditions. An alternative to dextran 10 is PVP 10, which may lead to lower, but still acceptable, mechanical properties (Table 1). PVP-based formulations are used in formulation development [14], [24]. Low molecular weight PVP (grades K12 and K17) are used as solubilizers in parenteral applications, which provide cryo-protection to the product and inhibit potential excipient crystallization [12]. Similarly, Ficoll® was mentioned to enhance glass transition temperature [14] and maltodextrin was identified as a cryoprotectant excipient for proteins [16], but these components are not available at an excipient grade compatible for parenteral route and other polymers may be preferred. As maltodextrin and CMC-based formulations did not exhibit the best physico-chemical results (F2 to F4 formulation, Table 1), PVP 10 or dextran 10 were selected for further vaccine applications. Finally, CMC was discarded to prevent potential high viscosity issues.

Sugars tested in this study gave comparable results. It was previously highlighted that maltose is prone to react with amino acid side chains of proteins (glucation, Maillard reaction) and that raffinose could crystallize with a significant loss of protein activity [25]. Among disaccharides, sucrose and trehalose should be preferred as they are widely used in commercial bio-products [11]. In its favor, trehalose presents the higher sugar glass transition temperatures and has also proved useful in preventing protein and virus degradation [26], [27]. In addition, a synergistic positive effect was observed when trehalose was mixed with albumin (HSA) to protect dengue virus in a vaccine candidate product [28], [29]. This combination was tested in F13 formulation but looked slightly less protective than the F16 formulation in terms of vYF infectious titer (Table 2). Note that a pure sucrose formulation was not able to prevent loss of infectious titer of vYF during freeze-drying process (data not shown), indicating that addition of other excipients is required to maintain infectivity of vYF.

Most live attenuated viruses are thermosensitive, losing approximatively 1.0 log_10_ after 1 week at 37 °C under freeze-dried form [30]. They often require complex formulations including numerous excipients [26] such as salts, polysaccharides, amino acids, cryoprotectants, tonicity modifiers and surfactants. Urea was also tested as a classical excipient for virus-based vaccines [31]. Following screening of excipients, calcium chloride, L-lysine, L-proline, P407, sorbitol and urea were identified as useful additives when mixed with trehalose and PVP to offer the best protection for vYF during the freeze-drying process and a thermal stress at 37 °C (F16 formulation, Table 2). Note that the main excipients added to stabilize vYF induced a negative impact on glass transition temperatures. However, trehalose and PVP could balance that effect and F16 composition led to acceptable values with Tg′ > −35 °C and Tg > 50 °C. Elevated Tg′ guarantees a low process time compatible with industrial constraints and a high Tg is required to maintain appearance of stable solid dried forms (to avoid collapse). Considering that the inherent temperature sensitivity of vaccines is particularly significant for live virus-based vaccines, elevated Tg exhibited by the selected formulation, F16, should help to stabilize vYF in both micropellet and freeze-dried forms. This is key for vaccine products, including yellow fever vaccines [32], as during vaccination campaigns in endemic zones in Sub-Saharan Africa and South America, they can be exposed to temperatures up to 40 °C just before vaccination, especially during the latter stages of transportation (cold chain break) [33]. In addition, it was shown that micropellets (F16 formulation) did not significantly change in terms of size parameters (diameter and polydispersity) and appearance during the accelerated stability study, even after several days at elevated storage temperatures (i.e. 2 weeks at 37 °C or 1 week at 45 °C, data not shown). With close kinetic parameters (activation energies, pre-exponential factors), two-step kinetic models were obtained for both solid forms, indicating complex degradation progresses. Same type of kinetic models were determined for both micropellets and conventional lyophilized forms (being relatively similar dried forms), and thus, reinforcing the pertinence of the type of kinetic models obtained here. In such a case, it was previously demonstrated that the application of wAIC and wBIC scores allowed discrimination of whether one or two-steps model is more likely to be correct [7], [8], [9], [34]. These criteria take into account not only the quality of fit, such as the sum of residual squares (RSS), but also the number of experimental points available and model parameters used. Additionally, they determine which model is more likely to be correct and quantify how much more likely. Hence, possible errors of over-fitting were avoided and it was statistically confirmed that the two-step models were required to describe the loss of virus infectivity. Two-step models (Eqs. (2), (3)) identified to describe loss of infectious titer of vYF for micropellets and lyophilized products are aligned with previous studies mimicking complicated decomposition of biological compounds [35], [36] and more particularly showing loss of virus infectivity (measles vaccine, ALVAC poxvirus vaccine, attenuated virus vaccine) in a bi-phasic way, with an initial rapid drop followed by a long gradual decrease phase [7], [30], [37]. It is critical to note at this point that these results do not have a real mechanistic basis, but it rather only depicts a phenomenological mathematical model applied for fitting the reaction course. Kinetic models determined in our study aimed to describe degradation progress of vYF by connecting time and temperature with Arrhenius-based relations. In practice, such kinetic models are useful as they were already used to accurately predict degradation level of bio-products when stored under recommended conditions (typically in the 2–8 °C range) or during excursions of temperature [9], [34]. Finally, <1.0 log_10_ CCID50 loss were experimentally observed for both micropellets and lyophilizates incubated for 14 days at 37 °C or for 3 months at 25 °C. Additionally, kinetic models indicated that <1.0 log_10_ CCID50 loss can be expected under standard storage conditions (i.e. 3 years at 5 °C), showing similar stability behavior for both solid forms. Note that this similar stability behaviour could also be assessed by the comparison of additional critical attributes of the virus, i.e. the size of the virus possibly determined by NTA (Nanoparticle tracking analysis) or by microscopic observations (TEM) and also the shape and integrity of the virus by the latter technic.

Predictive bands for infectious titer of vYF at 5 °C (Fig. 6, dashed lines) were larger for micropellets. The filling process was performed by weight for micropellets with 8% of variation (25 ± 0.2 mg per vial), inducing a variation of ±0.5 log_10_ CCID50 for an infectious titer targeted at 5.0 log_10_ CCID50. This observation was in full agreement with predictive band obtained by bootstrap method (Fig. 6, dashed lines) leading to confidence intervals of ±0.5 log_10_ CCID50 for predictions at 5 °C. By another hand, conventional lyophilized products were obtained following a filling by volume (0.3 mL per vial), inducing higher accuracy in terms of virus concentration. A bootstrap confidence interval (95% CI) of ±0.2 log_10_ CCID50 was obtained for predictions at 5 °C, aligned with analytical method accuracy for infectious titer determinations. In agreement with Mishra et al. [38], results presented in this study demonstrated that bootstrap method can advantageously be used to give accurate and realistic 95% confidence intervals for stability predictions.

Thermal sensitivity of vYF determined by infectious titer losses at elevated incubation temperatures (Fig. 5) is in agreement with VVM (Vaccine Vial Monitor) criteria [39] and WHO/UNICEF considerations in global immunization program for live-attenuated yellow fever vaccine that is classified as a medium stability product [40], with loss ≤1.0 log_10_ CCID50 when exposed for 14 days at 37 °C, 3 months at 25 °C and >3 years at 5 °C. Beyond such thermal sensitivity classifications and the use of VVM system, initially introduced as an indicator of heat induced deterioration in vaccines [32], kinetic models presented here (Eqs. (2), (3)) can advantageously be used to follow in real-time shelf-life of vaccines since the reaction rate equation is integrated into electronical time-temperature compact devices [41]. Thanks to integration of kinetic models into an Intelligent-Internet of Things (IoT) Outbound Logistics Knowledge Management (IOLMS) System [42], geo-localization of vaccines and their level of degradation can be known in real-time enabling emergence of modern supply chain management during shipments of vaccines. It would particularly be of major interest during last miles when vaccines cannot anymore be stored under the controlled cold chain (2–8 °C range).

## 5 Conclusion

A new Vero-cell yellow fever vaccine was used to identify and select a formulation of different excipients that meet the constraints of stabilizing the virus and of providing adequate mechanical properties for obtaining micropellets.

Taken together, results presented in this study demonstrated the potential for micropellets as a novel solid vehicle for vaccines that meet processability and stability requirements. The findings of this study demonstrated that the appropriate mechanical properties were obtained (such as high breaking force and low friability), which may be essential for the industrialization of storage and transport steps. Size homogeneity was also obtained; this parameter could be required to ensure flowability of the product during transfer and filling steps, and as a consequence to ensure potency compliance. The industrialization of the process steps will generate challenges and an additional work of development will be necessary (as example, micropellet manufacture at 10–100 L batch size, powder handling in sterile conditions, dispensing in low moisture conditions, filling accuracy). Fine tuning of this process could also lead to additional potential benefits, such as dosing on demand (single-dose *vs*. multi-doses), combinations of vaccines on demand (by world area for example), stockpile in case of emergencies and the opportunity for the product to be filled in all types of containers.
