# Supplementary material for: A spray freeze dried micropellet based formulation proof-of-concept for a yellow fever vaccine candidate
Source: Eur J Pharm Biopharm. 2019 Sep;142:334–43. doi: 10.1016/j.ejpb.2019.07.008 (PMC6727866; doi:10.1016/j.ejpb.2019.07.008)
Supplement: Supplementary data 1 [file mmc1.docx]

**Supplementary Table S1**

Comparison of kinetic models, including one-step (zero-, first-order) and two-step models for the infectious titer of vYF formulated under FSD micropellets and conventional lyophilized forms. Adjusted kinetic parameters (activation energy, E; pre-exponential factor, A; reaction order, n) and the statistical AIC and BIC weights, sum of residual squares RSS, number of parameters used in simulations were displayed.

**
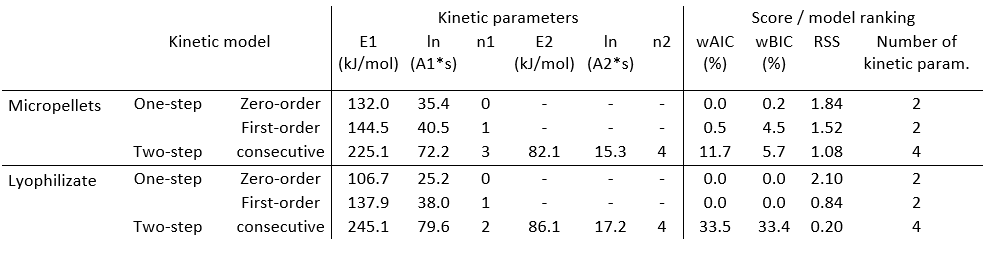
**
